# Testimonial of ecological and biogeographic patterns: parasite assemblages of deep water catsharks (Pentanchidae) in Icelandic waters

**DOI:** 10.1017/S0031182026101632

**Published:** 2026-03

**Authors:** Andrea Higueruelo, Bjoern C Schaeffner, Anna Soler-Membrives, Sara Dallarés

**Affiliations:** 1 Departament de Biología Animal, de Biología Vegetal i d’Ecologia, Universitat Autónoma de Barcelona, Barcelona, Spain; 2 Institute for Experimental Pathology at Keldur, University of Iceland, Reykjavík, Iceland; 3 Department of Anatomy, Physiology, and Pharmacology, School of Veterinary Medicine, St. George’s University, Saint George, Grenada

**Keywords:** deep water cat sharks, host–parasite interrelationships, metazoan parasites, North Atlantic Ocean, parasite ecology, Pentanchidae

## Abstract

Pentanchids (Elasmobranchii) are among the most species-rich groups of chondrichthyans. In the North Atlantic Ocean, the Icelandic catshark [*Apristurus laurussonii* (Saemundsson)], white ghost catshark (*Apristurus aphyodes* Nakaya & Stehmann), and mouse catshark [*Galeus murinus (Collett)*] are commonly found in deepwater habitats. However, information on their parasite communities remains scarce. This study provides the first comprehensive characterization of the metazoan parasite communities of the 3 pentanchid species. In total, 56 specimens were collected in Icelandic waters at depths of 466–1322 m between 2023 and 2024 and examined using standardized parasitological protocols, including morphological and molecular methods. Infection patterns were assessed in relation to size, maturity, body condition and capture area of hosts. Parasite intensities in all sharks ranged from 2 to 227 individuals, comprising 15 different taxa and resulting in 27 new parasite–host records, some of which likely representing new species. Eight out of 9 commonly found parasites did not display a high degree of host-specificity, indicating similar feeding habits, niche preferences, and trophic position of these sympatric species. Nonetheless, multivariate analyses revealed significant differences in the structure and composition of their parasite assemblages, with some parasites representing indicator species and occurring more abundantly and frequently in a certain deepwater catshark species. In addition, significant small-scale geographic differences were detected. At a broader geographical scale, North Atlantic pentanchids showed higher parasite richness and diversity, and lower dominance compared to standardized data from Mediterranean counterparts. Ecological factors underlying these patterns on host–parasite dynamics in (deepwater) cat sharks are discussed.

## Graphical abstract

**Figure:**
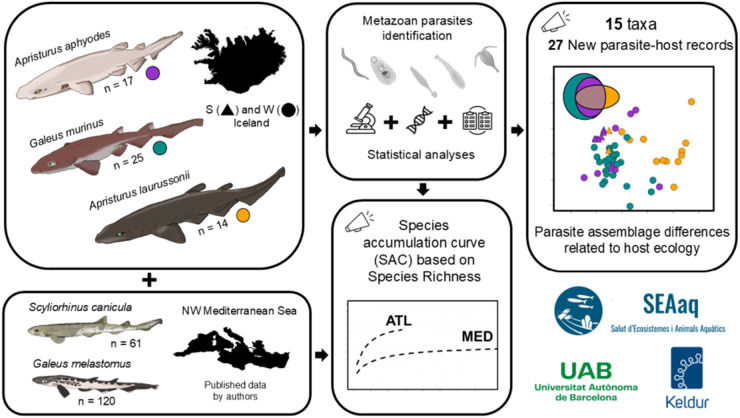

## Introduction

Catsharks are a diverse group of small, bottom-dwelling, generally non-migratory sharks characterized by their elongated, cat-like eyes, adapted for seeing in low light conditions (Compagno, 1984). They were originally classified within the family Scyliorhinidae. However, following a taxonomic revision that distinguished between catsharks and deepwater catsharks, the latter were transferred into a separate family, the Pentanchidae (Iglésias et al., 2005), now considered the largest family of living sharks. Despite the species richness, many pentanchid sharks are poorly known, most likely due to their life history in deepwaters, where research is still scarce but expanding (Ebert et al., 2021). Although the deep sea comprises over 90% of the World’s oceans and represents the largest biome on this planet, vast areas remain unknown and discovery rates of new species are high (Ramirez-Llodra et al., 2010; Selbach and Paterson, 2025). Despite the ecological significance and unique marine environment of Icelandic waters (North-East [NE] Atlantic Ocean), their marine biodiversity remains relatively understudied (Omarsdottir et al., 2013). In this region, 3 pentanchids, namely the Iceland catshark [*Apristurus laurussonii* (Saemundsson)], the white ghost catshark (*Apristurus aphyodes* Nakaya & Stehmann), and the mouse catshark [*Galeus murinus* (Collett)] are among the most frequent chondrichthyans (Jakobsdóttir et al., 2023). They are small (i.e. less than 80 cm in length), bottom-dwelling species distributed in the NE Atlantic Ocean (*A. laurussonii* shows the broadest distribution encompassing North and Central Atlantic waters) and found across a wide depth range in the continental slopes (between 380 and 2060 m, Ebert et al., 2021). Currently classified as ‘Least Concern’ in the International Union for Conservation of Nature’s (IUCN) Red List of Threatened Species, they are generally attributed stable population trends (*A. laurussoni* and *G. murinus*) (Iglésias, 2015; Walls, 2015; Kulka et al., 2020). Despite their abundance and ecological importance in North Atlantic waters, knowledge on their basic biology (i.e. diet, behaviour) and parasite infections, among others, remains limited.

Parasites represent a significant portion of living organisms (Poulin, 2014) and play a significant role in determining the structure of communities and ecosystems through interactions with their hosts, influencing their behaviour and fitness and ultimately regulating their populations (Price et al., 1986; Thomas et al., 1998; Wood et al., 2007). They are also useful bioindicators, being able to provide valuable information on their host species, such as trophic interactions and migration patterns (and thus habitat preferences based on prey availability) (Williams et al., 1992; Alarcos and Timi, 2013; Dallarés et al., 2017), or reveal responses of free-living populations and communities to environmental impacts (MacKenzie, 1999; Vidal-Martínez et al., 2010). They have been used for many decades as indicators of fish population stocks, to address host phylogenetic relationships (MacKenzie and Abaunza, 1998; Locke et al., 2013) and, more recently, to help assessing the effectiveness of protected conservation areas (Braicovich et al., 2021). Despite playing a vital role in marine ecosystems and constituting an important component of Ocean’s biodiversity, fish parasites have often been neglected in biodiversity and ecosystemic studies (Klimpel et al., 2006).

As for many other North Atlantic elasmobranch species, studies on parasite communities of Icelandic deepwater catsharks are almost entirely absent. For instance, only a single parasite species has been recorded from *A. laurussonii* and *A. aphyodes*, the cestodes *Ditrachybothridium macrocephalum* Rees, 1959 and *Yamaguticestus kuchtai* Caira, Pickering & Jensen, 2021(Bray and Olson, 2004; Caira et al., 2021), respectively, while parasite records from *G. murinus* are entirely lacking.

In contrast, there are a considerable number of studies on different ecological aspects of the 2 most common catsharks distributed not only in the Atlantic Ocean but also in the Mediterranean Sea, namely the blackmouth catshark (*Galeus melastomus* Rafinesque) and the small-spotted catshark [*Scyliorhinus canicula* (L.)] (Massutí and Moranta, 2003; Follesa et al., 2019). Their parasite community is particularly well-known and characterized, with 20 and 29 parasite species, respectively, reported across their respective distribution ranges, including monogeneans, cestodes, trematodes, nematodes, copepods and isopods (see Pollerspöck and Straube, 2025 for a complete list of references). In the Balearic Sea alone, 15 and 12 parasite species have been reported infecting *G. melastomus* and *S. canicula*, respectively (Dallarés et al., 2017; Higueruelo et al., 2024). Given the relatively high diversity of parasites found in Mediterranean catsharks, and considering the higher biomass, species richness and abundance of deep-sea fish assemblages in the Atlantic (Massutí et al., 2004), it is likely that North Atlantic catsharks will reveal broader parasite communities with potentially new species yet to be discovered.

Although molecular ecology and the use of genetic tools are still poorly applied in parasitology compared to free-living organisms (Selbach et al., 2019), molecular tools are of great interest for addressing parasite species identification and host specificity (Criscione et al., 2005). These tools are highly advisable for the characterization of parasite assemblages, where larval forms (impossible to identify solely based on morphological features), cryptic species and phenotypic plasticity frequently occur. Therefore, combining traditional parasitological techniques based on morphology with molecular analyses is the most effective approach for studying parasite communities across different ecological and geographical contexts. In addition, the use of ecological indices, such as species richness, diversity or dominance, is also widely applied in studies of parasite communities, providing important ecological insights.

Parasitological investigations play a critical role in deepening our understanding of biodiversity and the complex interactions within marine environments. In order to broaden the available knowledge on parasite infection patterns in catsharks from an ecological perspective, the parasite communities infecting *A. laurussonii, A. aphyodes* and *G. murinus* from Icelandic waters were characterized and described for the first time in the present study. In addition, differences among these parasite assemblages as a function of different factors (e.g. host species, host maturity, area of capture) were assessed and parasitological descriptors and infection patterns were analysed jointly with data from Mediterranean catsharks and discussed from an ecosystemic approach.

## Materials and methods

### Study area and sample collection

**Figure 1.**
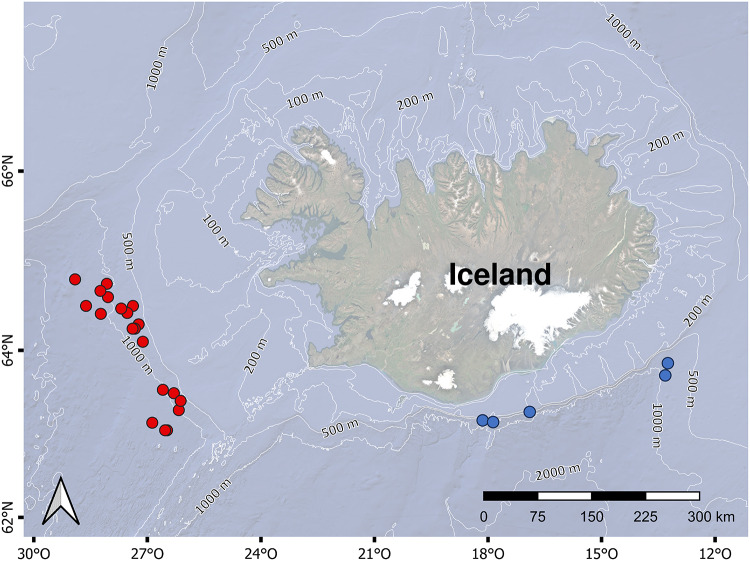
Map of the study area. Dots indicate the sampling stations where the pentanchid sharks were collected. Red dots: western area; blue dots: southern area. Figure 1 long description.

A total of 17 specimens of *A. aphyodes*, 14 *A. laurussonii* and 25 *G. murinus* were collected in autumn of 2023 and 2024 at depths ranging between 466 and 1322 m (Table 1) in southern and western Icelandic waters (North Atlantic Ocean). Samples were collected in the frame of the annual Icelandic Autumn Groundfish Surveys from the Marine and Freshwater Research Institute Iceland (MFRI) on board of the research vessels Árni Friðriksson and Breki. For comparative analysis, the sampling stations were divided into southern and western areas (Figure 1), and the number of individuals caught from each area is presented in Table 3.

**Table 1.** Biometric data of *Apristurus aphyodes, Apristurus laurussonii* and *Galeus murinus* sampled in Icelandic waters Table 1 long description.

	*A. aphyodes*	*A. laurussonii*	*G. murinus*
*N* (*)	17 (9)	14 (2)	25 (21)
Maturity (%)	94.1	92.9	68.0
Depth (m)	745 (233)	1112 (159)	852 (197)
	546−1,278	796−1281	466−1322
TL (cm)	60.9 (7.8)^a^	67.3 (3.7)^a^	49.5 (6.5)^b^
	51.5−71.0	61.5−75.3	30.5−54.9
TW (g)	798.4 (206.9)	941.3 (165.0)	453.5 (141.4)
	472.0−1086.0	672.0−1180.0	91.5−622.2
Kn	1.01 (0.11)	1.01 (0.12)	0.97 (0.07)
	0.84−1.31	0.76−1.17	0.83−1.11

Different superscript letters indicate statistically significant differences among host species.

*N*: sample size; Maturity: Percentage of sexually mature individuals. Mean values followed by standard deviation and range values (minimum – maximum) of depth of collection, total length (TL), total weight (TW) and Le Cren relative condition index (Kn)

(*): Number of females.

Immediately upon capture, a photograph of each individual was taken and records of total length (TL, in cm), total weight (in g) and sex were obtained for each shark individual. Five spiral valves from *A. aphyodes* were immediately preserved in 95% EtOH and 5 and 4 spiral valves, from *A. laurussonii* and *A. aphyodes*, respectively, were preserved in 4% buffered formalin for molecular and morphological parasite identification purposes. Specimens were frozen at − 20 °C for further examination.

### Dissection procedure and parasitological study

Prior to dissection, the external surfaces of each individual were examined macroscopically for ectoparasites. After removal of the abdominal organs (i.e. liver, gonads, stomach, spiral valve, spleen and pancreas), which were preserved separately for further examination, the eviscerated weight (EW) was recorded. Subsequently, the remaining organs (i.e. nostrils, gills, heart, kidneys and brain) were also removed. Maturity was inferred from the overall appearance of reproductive organs, the degree of clasper calcification in males, and the presence of egg capsules in females (Higueruelo *et* *al.,* 2025).

All organs were examined for metazoan parasites under a stereomicroscope. In 9 individuals (3 *A. aphyodes* and 6 *A. laurussonii*), the liver or gonads were discarded on board for reasons beyond the authors’ control and were therefore unavailable for examination or inclusion in subsequent analyses. Mouth and abdominal cavity were washed with 0.9% saline solution to recover detached parasites potentially present in these cavities. The musculature between pectoral and caudal fins was cut into thin slices and thoroughly inspected for potential encysted endoparasites. All recovered parasites were counted and stored in 70% ethanol.

For morphological identification, platyhelminths were stained either with Delafield’s haematoxylin or iron acetocarmine, dehydrated through a graded series of ethanol, cleared in clove oil and permanently mounted in Canada balsam on microscope slides. Nematodes were examined as semi-permanent mounts in pure glycerine. All parasites were identified to the lowest possible taxonomic level. Parasite identification was based on dichotomic keys and specialized bibliography (mainly the monographs – Kabata, 1979; Moravec, 1994, 2001; Palm, 2004).

Parasites selected for molecular identification were preserved in 95% EtOH in the freezer. When possible, hologenophores (sensu Pleijel et al., 2008) were prepared. When specimens were too small, a morphologically identical voucher was selected. Representative voucher specimens were deposited in the parasitological collection of the Zoology unit of the Universitat Autònoma de Barcelona (Barcelona, Spain) (Accession numbers: M3–M8, C47–C51 and D9–D10).

Genomic DNA was extracted using a QIAgen DNA extraction kit or a QIAcube HT system, following the manufacturer’s protocol. Mitochondrial cytochrome oxidase 1 (mtCOI) and internal transcribed spacer (ITS) for nematodes and partial nuclear large subunit ribosomal DNA (28S rDNA) for the rest of parasites were amplified by polymerase chain reaction (PCR) amplifications. These were performed as described in Constenla et al. (2014) or Brabec et al. (2012), respectively, adjusted for Taqman Expression mastermix. The PCR-products were analysed by capillary electrophoresis using a High-Resolution DNA kit in a Qiaxcel Advanced Instrument and viewed in the Qiaxcel ScreenGel (Qiagen) or analysed on RedGel-stained 1% TAE agarose gels. Sequencing of PCR products was performed by Genewiz-Azenta or Macrogen Inc. using either the Sanger method or capillary electrophoresis, respectively. Obtained sequences were aligned using BioEdit 7.7.1 (Hall, 1999) checked visually for accuracy and compared to available sequences in GenBank with Mega v.11 (Tamura et al., 2021).

### Data analysis

Parasite prevalence (P), mean abundance (MA), mean species richness (MSR) and species richness (S) were calculated for each host species, grouped by area, following Bush et al. (1997). A 95% confidence interval for the mean abundance was calculated with the software Quantitative Parasitology (QPweb) (Reiczigel et al., 2019). Parasite diversity was estimated by Brillouin’s index (*H*) and calculated with PRIMER 6 software (Anderson et al., 2008). The Berger-Parker dominance index (B-P dom) was calculated as the proportion of individuals belonging to the most abundant parasite species relative to the total number of parasites in each individual host. Le Cren’s relative body condition index (Kn) was calculated separately for each shark species with the formula Kn = EW/(α × *TL*β), where α and β are the slope and the intercept of the weight–length relationship, of the entire dataset of sampled fish (Le Cren, 1951). Parasite taxa with a prevalence <5% in all hosts were considered accidental, while parasite taxa with >25% prevalence in at least 1 host species were considered common.

Fish biometric data (TL and Kn) and parasite infection parameters were tested for normality and homoscedasticity using the Shapiro–Wilk test and Levene’s test, respectively. Data distribution was also plotted for visual assessment. When necessary, variables were log or square root transformed to comply with normality and homoscedasticity requirements for parametric tests.

To detect potential associations in each host species between individual fish biological data and parasitological descriptors (i.e. richness, total abundance, abundance of common parasite taxa and diversity), Pearson’s or Spearman’s correlation tests (the latter when normality was not satisfied) were used. Since the Western area had the highest number of specimens, interspecific differences in parasitological descriptors, parasite abundance, and parasite prevalence were evaluated using only individuals from this area. Differences among the 3 host species were tested using ANOVA for parametric data and Wilcoxon or Kruskal–Wallis tests for non-parametric data, with post hoc pairwise comparisons performed using TukeyHSD and Dunn’s tests (Bonferroni- or Holm-adjusted), respectively. Additionally, Fisher exact test and subsequent pairwise comparisons using the function *pairwiseNominalIndependence* were employed to assess differences in the prevalence of common parasites among host species.

Whenever sample size was high enough (with at least 8 individuals in each group), these potential differences were also tested between immature and mature individuals (for *G. murinus*) and between western and southern sampling areas (for *A. aphyodes*). Intraspecific differences among areas were assessed using TL as a covariate, employing generalized linear models (GLMs) or analysis of covariance (ANCOVA). Distributions were selected according to data type: Poisson for count data (e.g. S), binomial with a logit link function for prevalence data, and Gaussian or Gamma for parametric and non-parametric variables, respectively.

Ordination of parasite infracommunities (i.e. all parasite taxa infecting a given individual host) according to the different hosts and sampling areas was visualized with a non-metric multidimensional scaling (nMDS) based on a Bray–Curtis dissimilarity matrix calculated from log + 1 transformed species abundance data. An Euler diagram was also constructed to illustrate the amount of specific or shared parasite taxa among host species. A PERMANOVA (permutational analysis of variance) was conducted using parasite abundance and prevalence data (using a Bray–Curtis and Jaccard dissimilarity matrix, respectively) to reveal potential differences in the composition and structure of parasite assemblages across hosts and areas (the latter only for *A. aphyodes*). PERMANOVA analyses were performed using the *Adonis2* function, followed by pairwise tests, with 999 unrestricted permutations of raw data. The Indicator Value Index (IndVal) (Dufrêne and Legendre, 1997) was then applied to identify the most representative parasite species for each host species and of each area in the case of *A. aphyodes*.

Previously published data by present authors on parasite infection parameters of the 2 most common Mediterranean catsharks (i.e. *S. canicula* and *G. melastomus*) (Dallarés et al., 2017; Higueruelo et al., 2024) were used to explore large-scale geographic patterns, something possible because parasitological protocols matched those followed in the present study. Differences on parasitological indices (i.e. MA, S, H, B-P dom and MA of each parasite phylum) between Mediterranean and Atlantic catsharks were tested with Wilcoxon and Pearson’s Chi-squared test. Species accumulation curves (SACs) were used to predict and compare total species numbers for each host and study area using the *specaccum* function with random method and 999 permutations. For SACs, only non-accidental parasites were considered. Statistical analyses were conducted with R version 4.4.1. Correlations were considered strong when the correlation coefficient (*R*) was higher than 0.65. Statistical significance was set at 0.05.

## Results

A total of 56 pentanchid shark individuals were examined for parasites, 82.1% of them being sexually mature. Overall, sharks TL ranged between 30.5 and 75.3 cm, with *G. murinus* being significantly smaller than *Apristurus* species (K-W, χ^2^ = 33.92, *P* < 0.001) (Table 1).

### Parasite composition and parasitological descriptors of Icelandic pentanchids

A total of 2780 metazoan parasites belonging to 15 different taxa were recovered from the 3 analysed shark species, including 1 nematode, 1 cirriped, 2 copepods, 4 monogeneans, 5 cestodes and 2 digeneans (Table 2). These findings represent 27 new parasite–host records. All sharks were parasitized by at least 1 parasite, with parasite abundance ranging from 2 to 227.

**Table 2.** Descriptors of parasite component populations (i.e. All parasites of a given species infecting a given host population) on the 3 pentanchid species captured off Iceland Table 2 long description.

				*A. aphyodes* (West)		*A. aphyodes* (South)		*A. laurussonii* (West)		*A. laurussonii* (South)		*G. murinus* (West)	
Parasite taxa		Stage	Infection site	P%	MA (95% CI)	P%	MA (95% CI)	P%	MA (95% CI)	P%	MA (95% CI)	P%	MA (95% CI)
**Nematoda**													
	*Anisakis* Type I*	L3	BC, GO, K, L, M, P, S, SP, SV	100^A^	28.3^a^ (14.3−47 .2)	100	68.9 (41.6−94.8)	50.0^B^	4.83^b^ (1.25−14.2)	100	71	100 ^A^	45.7^a^ (33.2−64.8)
**Crustacea**													
Thecostraca	*Anelasma squalicola*	A	SK, L									12.0	0.1 (0−0.2)
Copepoda	*Lernaeopodina* sp.	A	G									4.0	0.04 (0−0.1)
	Taeniacanthidae gen. sp.	A	N									4.0	0.04 (0−0.1)
**Platyhelminthes**													
Monogenea	*Calicotyle* sp.*	J, A	BC, SP	77.8^A^	3.9^a^ (1.3−7.8)			16.7^B^	0.2^b^ (0−0.3)			32.0^B^	1.0^b^ (0.4−1.9)
	Cathariotrematinae gen. sp.*	J, A	N					83.3	5.3 (3.2−7.6)				
	Hexabothriidae gen. sp.*	J, A	G	55.6^A^	3.6 (1.3−6.1)			83.3^AB^	3.3 (1.8−5.3)	50	1.5	96.0^B^	5.7 (4.1−8.1)
	*Squalotrema* sp.	J, A	N									16.0	0.2 (0−0.3)
Cestoda	Ditrachybothridium macrocephalum*	A	SV	66.7	3.6 (1.0−7.8)							48.0	4.2 (1.9−8.5)
	*Grillotia* sp*	Ps	M	44.4	0.6 (0.1−1.0)							36.0	0.8 (0.3−2.2)
	Hepatoxylon trichiuri*	Pd	GO, L, S	22.2	0.3 (0−0.9)			8.3	0.1 (0−0.3)			32.0	0.6 (0.2−1.0)
	*Heterosphyriocephalus tergestinus*	Pd	S									4.0	0.04 (0−0.1)
	Yamaguticestus kuchtai*	A	SV	44.4	7.6 (1.7−23.9)			50.0	0.8 (0.2−1.2)	50	0.5	12.0	0.1 (0−0.2)
Trematoda	Otodistomum cestoides*	Mt	S	22.2	0.2 (0−0.4)							48.0	0.6 (0.1−2.3)
	Digenea indet.	A	BC					16.7	0.2 (0−0.3)				

Different superscript lowercase and capital letters show significant differences in the mean abundance and prevalence, respectively, of parasite populations among host species from West area.

* Parasites considered common in the present study (>25% prevalence in at least 1 host species).

Developmental stage, location within host, prevalence (P %) and mean abundance (MA, followed, in parentheses, by a 95% confidence interval when *N* > 2) are provided for the parasites found in *Apristurus aphyodes, Apristurus laurussonii* and *Galeus murinus*. For *A. Aphyodes*, values are also presented separately for 2 areas of capture: west and south of Iceland.

Abbreviations for infection sites within host: BC, body cavity; G, gills; GO, gonad; K, kidney; L, liver; M, muscle; N, nostrils, P, pancreas; S, stomach; SK, skin; SP, spleen and SV, spiral valve. Abbreviations for developmental stages: A, adult; J, juvenile; L, larvae; Mt, metacercaria; Pd, plerocercoid; Ps, plerocercus.

Among the recovered parasites, 5 taxa were found in all 3 analysed hosts. *Anisakis* Type I (sensu Berland, 1961), found as third stage larvae encysted in several organs and displaying the highest prevalence (89.3% overall prevalence) was identified as *Anisakis simplex* (Rudolphi, 1809) in the 3 hosts (GenBank accession numbers: PV933132, PX101432). Shared monogeneans consisted of a yet undescribed species of *Calicotyle* Diesing, 1850 infecting the rectum, and a potentially new species of the family Hexabothriidae infecting the gills (GenBank accession numbers: PV972204, PV972205, PV972206), with overall prevalences of 30.4% and 71.4%, respectively. Plerocercoids of the cestode *Hepatoxylon trichiuri* (Holten, 1802) (GenBank accession number: PV972208) were found encysted in the gonad, liver and stomach wall with a prevalence of 19.6%, while adult specimens of *Yamaguticestus kuchtai* (Caira et al., 2021) (GenBank accession numbers: PV972202, PV972203) were found infecting the spiral valves in 25% of examined sharks.

In *A. aphyodes* and *G. murinus*, trypanorhynch plerocerci and a metacercariae encysted in the tail musculature and stomach wall, respectively, were also commonly found and genetically identified as *Grillotia adenoplusia* (GenBank accession number: PV972201) and *Otodistomum cestoides* (Van Beneden, 1870) (GenBank accession number: PV972207).

In total, 8 parasite taxa were found in *A. aphyodes*, none of which were exclusive to this species (Table 2). The most prevalent and abundant parasite was *Anisakis* Type I. The yet undescribed species of *Calicotyle* (*Calicotyle* sp.) showed the highest prevalence and abundance in this host, with up to 13 parasites found in a single shark individual. For this host, a strong positive correlation between Berger–Parker dominance index and fish TL was found (rho = 0.91, *p* < 0.001) while parasite richness and Brillouin’s index were negatively associated with fish TL (rho = – 0.79 and −0.70, *p* < 0.002).

In *A. laurussonii*, the most prevalent parasites were the monogeneans Hexabothriidae gen. sp. and Cathariotrematinae gen. sp. (GenBank accession number: PV972210); the latter found infecting the nostrils and exclusively in this species. The abundance of *Anisakis* Type I in *A. laurussonii* was positively correlated with fish TL (rho = 0.71, *p* = 0.005) and parasite richness with fish Kn (rho = 0.67, *p* = 0.008).

All examined specimens of *G. murinus* were infected with *Anisakis* Type I, and all but 1 individual with Hexabothriidae gen. sp. These were the 2 most abundant parasites in this host, reaching maximum abundances of 182 and 19 parasites, respectively. Five parasite taxa were exclusive to *G. murinus*: the monogenean *Squalotrema* sp., the copepods *Lernaeopodina* sp. and Taeniacanthidae gen. sp., the cestode *Heterosphyriocephalus tergestinus* (Pintner, 1913) and the cirriped *Anelasma squalicola* Darwin, 1852 (GenBank accession number: PV972209). The latter species was typically found externally attached near the mouth and, interestingly, in 1 case the parasite had perforated the skin and was found in the liver. TL of *G. murinus* was positively correlated with parasite total abundance and with abundance of *Anisakis* Type I (rho = 0.65 and 0.70, *p* < 0.001). Concordantly, significant differences in the same 2 parasitological descriptors were observed between juvenile and adult host specimens, with higher values found in mature individuals (*t*-test, *t* = −3.73 and *t* = −4.06, respectively, *p* < 0.003 in both cases). Parasite assemblages of adult sharks also displayed a higher dominance index (Wilcoxon test, *W* = 30.50, *p* = 0.031).

### Host-related and geographical patterns of parasite communities of Icelandic pentanchid sharks

**Figure 2.**
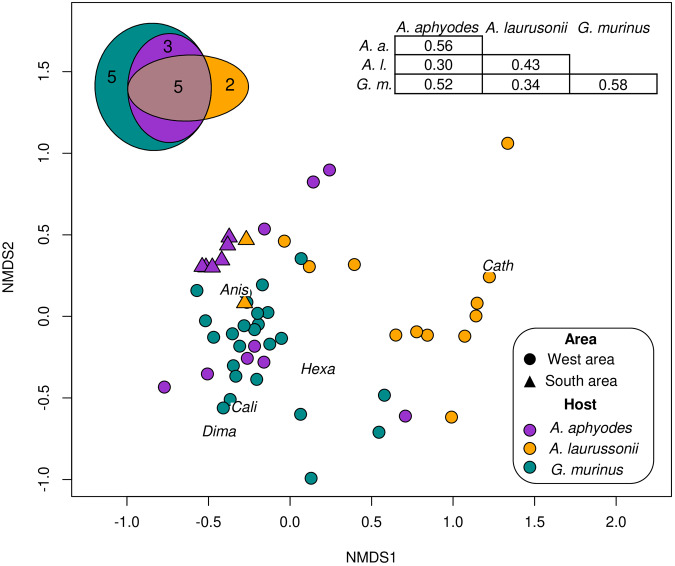
Non-metric multidimensional scaling (nMDS) illustrating the ordination of parasite assemblages according to host species and area of capture. The analysis is based on a Bray–Curtis dissimilarity matrix calculated from log-transformed (log (*x* + 1)) parasites abundance data. A Bray–Curtis similarity matrix displaying mean similarities within/among hosts (top right) and an Euler diagram depicting distribution of parasite taxa across hosts (top left) are also shown. Abbreviations for representative species according to Indicator value analyses are shown in the plot: Anis, *Anisakis* Type I; Cali, *Calicotyle* sp.; Cath, Cathariotrematinae gen. sp.; Dima, *Ditrachybothridium macrocephalum*; Hexa, Hexabothriidae gen. sp. Figure 2 long description.

The nMDS ordination plot based on parasite abundance data (stress = 0.167) showed a grouping pattern based on host identity (Figure 2). The most similar intraspecific parasite assemblages were those of *G. murinus* and *A. aphyodes*, which showed highest mean intraspecific Bray–Curtis similarity indices. Regarding interspecific comparisons, *A. aphyodes* and *G. murinus* displayed the most similar assemblages while *A. laurussonii* assemblages were the most differentiated. PERMANOVA analyses revealed significant differences in both the structure (Bray–Curtis similarity index, *F* = 10.70, *p* < 0.001) and composition (Jaccard similarity index, *F* = 12.67, *p* < 0.001) of parasite communities among the 3 hosts. Subsequent pairwise comparisons confirmed that these differences were present across the 3 host species (*F* = 4.69–14.93, *p* < 0.003 in all cases). The Euler diagram (Figure 2) illustrated that out of the 15 parasite taxa identified, 7 were exclusively found in 1 host, 6 of these classified as uncommon or accidental (P < 25%). The indicator value analysis identified Cathariotrematinae gen. sp. as strongly associated with its single host *A. laurussonii* (IndVal = 0.71, *p* = 0.001) while Hexabothriidae gen. sp and *D. macrocephalum* were moderately associated with *G. murinus*, indicating that they occur more frequently and abundantly in this species (IndVal = 0.52, *p* = 0.003 and IndVal = 0.33, *p* = 0.045, respectively). No significant indicator species were detected for *A. aphyodes.*

The comparison among individuals from the West area revealed that total parasite abundance was lower in *A. laurussonii* compared to the other 2 hosts (ANOVA, *F* = 8.92, *p* < 0.001). Similarly, the abundance and prevalence of *Anisakis* Type I was significantly lower in *A. laurussonii* (K-W, χ^2^ = 17.29, *p* < 0.001; Fisher test, *p* < 0.001). Additional differences in the prevalence and abundance of other parasite taxa are detailed in Table 2. No significant differences were found between pentanchid parasite communities with respect to MSR, B-P dom or H.

In the case of *A. aphyodes*, geographic differences in parasite assemblages were identified. The nMDS showed a separation between samples caught from the southern and western areas of Iceland (Figure 2). Parasite communities of catshark individuals from the southern region appeared more tightly clustered together (Bray–Curtis similarity index = 61%) compared to the more dispersed communities in the western region (Bray–Curtis similarity index = 36%), although the overall similarity between both areas was only slightly higher (Bray–Curtis similarity index = 39%). There were significant geographical differences on the abundance and presence of parasites according to PERMANOVA analyses (PERMANOVA, *F* = 9.25 and *F* = 22.20, respectively, *p* < 0.001 in both cases). The indicator value analyses associated *Calicotyle* sp., *D. macrocephalum* and Hexabothriidae gen. sp. with sharks from the western area (IndVal = 0.78, 0.67 and 0.56; *p* < 0.026 in all cases) and *Anisakis* Type I with those of the southern area (IndVal = 0.71, *p* = 0.030). Despite the lack of differences in total parasite abundance (*p* > 0.05), southern pentanchids exhibited lower MSR (GLM, *p* < 0.029) and *H* (ANCOVA, *p* = 0.002) and a higher B-P dom as well as *Anisakis* Type I abundance (ANCOVA, *p* < 0.001 and *p* = 0.03, respectively).

### Large-scale geographic comparison of catshark parasite assemblages

When comparing the parasite assemblages of the most common Mediterranean and Atlantic catsharks, no significant difference in total parasite abundance was found among hosts (*p* > 0.05). However, when considering parasites grouped by phylum, nematodes were more abundant and prevalent in hosts from the Atlantic Ocean (Wilcoxon test, *W* = 7863; Chi-squared, χ^2^ = 43.7, respectively, *p* < 0.001 in both cases), while crustaceans showed a higher abundance and prevalence in those from the Mediterranean Sea (Wilcoxon test, *W* = 3852, *p* < 0.001; Chi-squared, χ^2^ = 10.13, *p* = 0.001, respectively). In the case of platyhelminths, no significant differences were observed among hosts of both regions in terms of prevalence and abundance. Differences in parasitological indices were also found to be significant, with Atlantic catsharks displaying a lower B-P dom and a higher *H* and MSR (Wilcoxon test, *W* = 2460.5, 8071 and 7711.5, respectively; *p* < 0.001 in all cases) (Table 3).

**Table 3.** Descriptors of parasite component communities on the 3 pentanchid species captured off Iceland Table 3 long description.

	Iceland (present study)					Mediterranean Sea (Dallarés et al., 2017; Higueruelo et al., 2024)	
	*A. aphyodes* (West)	*A. aphyodes* (South)	*A. laurussonii* (West)	*A. laurussonii* (South)	*G. murinus* (West)	*G. melastomus*	*S. canicula*
N	9	8	12	2	25	120	61
S	8	1	7	3	13	15	12
MSR	4.3 (3.4−5.3)	1	3.1 (2.3−3.8)	2	4.1 (3.5−4.7)	1.7 (1.5−1.9)	2.4 (2.2−2.6)
TMA	48.0 (5.2−69.4)	68.9 (34.9−102.8)	14.5 (7.7−21.3)	73	59.1 (40.0−78.2)	40.8 (30.9−50.8)	55.2 (43.9−66.4)
H	0.72 (0.56−0.88)	0	0.63 (0.47−0.79)	0.11	0.59 (0.48−0.70)	0.15 (0.11−0.18)	0.30 (0.25−0.36)
B-P dom	0.68 (0.57−0.80)	1	0.63 (0.54−0.74)	0.97	0.75 (0.69−0.82)	0.93 (0.90−0.95)	0.87 (0.84−0.90)

Total richness (S), mean species richness (MSR), total mean abundance (TMA), Brillouin Diversity Index (*H*) and Berger–Parker Dominance Index (B-P dom) are displayed for parasite assemblages characterized in sharks captured off Iceland (*Apristurus aphyodes, Apristurus laurussonii* and *Galeus murinus*) and in the Balearic Sea (*Galeus melastomus* and *Scyliorhinus canicula*) (Dallarés et al., 2017; Higueruelo et al., 2024). Values are presented separately for the 2 areas of capture: west and south of Iceland. *N* = number of individuals. 95% Confidence interval is shown in brackets for MSR, TMA, *H* and B-P dom when *N* > 2.

**Figure 3.**
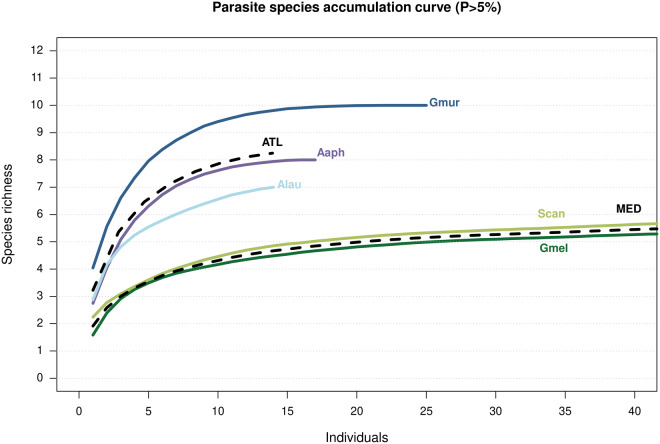
Species accumulation curves showing accumulation of parasite species by host and region. Hosts (solid lines): Aaph, *Apristurus aphyodes*; Alau, *Apristurus laurussonii*; Gmel, *Galeus melastomus*; Gmur, *Galeus murinus*; Scan, *Scyliorhinus canicula*. Regions (mean values, dashed lines): ATL, Atlantic Ocean; MED, Mediterranean Sea. Figure 3 long description.

The greater parasite richness occurring in the Atlantic Ocean compared to the Mediterranean Sea was reflected in the SACs shown in Figure 3. In general, the curves followed a typical accumulation pattern, with a steep initial increase that gradually flattened, although the curve associated with *A. laurussonii* did not stabilize. The 3 catshark species sampled in the Atlantic Ocean showed steeper slopes than those from the Mediterranean Sea.

## Discussion

This is the first study characterising the parasite communities of deep water catsharks from North Atlantic waters. Present findings provide valuable data on the parasite assemblages of 3 of the most common Icelandic pentanchids, uncovering 27 new host–parasite records and providing baseline data for future research on parasite ecology and environmental parasitology, 2 especially growing fields in the context of global change (Palm and Mehlhorn, 2011; Poulin, 2021; Sures et al., 2023).

The 3 pentanchid species assessed herein hosted relatively diverse and abundant parasite communities, dominated by generalist taxa. This is consistent with previous observations, in which parasite diversity decreases with depth but increases again near the sea floor (Marcogliese, 2002). The wide depth range, combined with a diverse diet, enables benthodemersal species to harbour a species-rich parasite fauna, especially compared to meso- and bathypelagic fish (Klimpel et al., 2006, 2009, 2010).

New findings concerning the only 2 previously recorded parasites in these 3 catshark species are reported. *Ditrachybothridium macrocephalum* had only been recorded in *A. laurussonii* in its plerocercoid form (Bray and Olson, 2004). However, in the present study, mature specimens were identified infecting *A. aphyodes* and *G. murinus*, supporting the hypothesis proposed by Faliex *et al.* (2000) that deepwater catsharks serve as definitive hosts for species of *Ditrachybothridium* Rees, 1959. The second previously reported parasite, *Y. kuchtai*, recently described in *A. aphyodes* as its type host (Caira et al., 2021), was also found in *A. laurussonii* and *Galeus murinus*. Therefore, the known host range is expanded for both cestode species.

Plerocercoids found in the tail musculature of *A. aphyodes* and *G. murinus* were tentatively identified as *G. adenoplusia* (Pinter, 1903) based on molecular results, which indicated conspecificity with *Grillotia* larvae from the Balearic Sea that had been identified as *G. adenoplusia* based on the study of oncotaxis (Dallarés et al., 2017; Isbert et al., 2023). Molecular characterization of adult specimens of this parasite, which will allow confirming unequivocally its identity, remains to be done. The definitive host of this parasite is known to be the bluntnose sixgill shark, *Hexanchus griseus* (Bonnaterre), a widely distributed species and capable of long-distance migrations (Ebert and Stehmann, 2013). This finding, together with the genetic structure population results of Vella and Vella (2017), who found shared haplotypes in *H. griseus* from the NE Atlantic and central Mediterranean Sea, suggests potential connectivity between the Atlantic and Mediterranean populations. Similarly, *Hepatoxylon trichiuri* has been reported in both the Atlantic and Pacific Oceans (Palm, 2004), while *O. cestoides* is known from the Atlantic Ocean and the Mediterranean Sea (Pollerspöck and Straube, 2025). These parasites use various large elasmobranchs as definitive hosts (Pollerspöck and Straube, 2025 and references therein). The frequent occurrence of these larval forms in Icelandic pentanchids suggests predation of these catsharks by larger sharks, a frequent phenomenon (Ebert, 1994; Dedman et al., 2024) that suggests intricate trophic interactions in the region that remain to be fully understood. *Anelasma squalicola* is a cirriped barnacle that directly extracts nutrients from its shark host, a trait that has drawn scientific interest (Rees et al., 2014, 2019; Ommundsen et al., 2016; Sabadel et al., 2022). Rees et al. (2019) concluded that this unique feeding strategy, described as a ‘de novo innovation’, triggered its global expansion, occurring so quickly that it didn’t have time to evolve into separate species. Molecular data from the present study, showing conspecificity with previously published sequences, further support this hypothesis by extending the known host range of *A. squalicola* to include *G. murinus*, and its geographic distribution northward into Icelandic waters. In addition, a barnacle was found inside the shark’s body cavity for the first time, where it was attached to the liver after penetrating the skin. Yano and Musick (2000) reported that *A. squalicola* is able retard the development of the reproductive organs of male sharks. Further studies monitoring this intriguing parasite and its potential effects on shark health would be welcome, especially considering its apparent rapid expansion.

The higher parasite loads, particularly of *Anisakis* Type I, found in mature individuals, together with the correlation observed between TL and parasite abundance, is consistent with the life cycle of this species. Indeed, *Anisakis* species, like many other parasites’ larval forms, accumulate throughout the lifespan of their paratenic or intermediate host (Mattiucci et al., 2017), potentially becoming more dominant over time. The life cycle of anisakid nematodes involves aquatic invertebrates as first intermediate hosts and cephalopods and fishes as second or paratenic hosts (Klimpel et al., 2004). Fishes, due to their longer lifespans and trophic positions, are more likely to carry *Anisakis* larvae compared to smaller, shorter-lived organisms such as crustaceans (Münster et al., 2015). Thus, the present findings may reflect an ontogenic shift of adult sharks towards higher-trophic-level prey items and a more diversified diet; patterns also reported in other cat sharks (Carrassón et al., 1992; Van der Heever et al., 2020). Nonetheless, such a diet shift is usually associated with a higher parasite richness (Poulin, 2004; Timi and Lanfranchi, 2013), which was not observed herein. To reliably detect dietary patterns, studies with a broader size range and a larger sample size would be necessary.

Regarding monogeneans, Cathariotrematinae gen. sp. and *Squalotrema* sp. were exclusively found infecting the nostrils (also referred to as the olfactory bulbs) of *A. laurussonii* and *G. murinus*, respectively. Both species belong to Cathariotrematinae, a monophyletic group of monocotylids known to parasitize shark nostrils (Bullard et al., 2021) and reported herein for the first time from pentanchid sharks. Contrary to the general believe that monogeneans were highly specific taxa (Poulin, 1992), there is growing evidence that various monocotylid species exhibit low host specificity (Chisholm and Whittington, 1996; Kritsky et al., 2017; Bullard et al., 2021). This contradicts present findings, according to which closely related parasite species sampled from the same area show increased host specificity in the nostrils.

Research on specific parasite groups often leads to selective necropsy practices (e.g. cestode-focused studies specifically targeting the spiral intestine) (Caira and Healy, 2004). While this kind of studies are clearly justified from a taxonomically-based approach, they can also leave the full parasite diversity in elasmobranchs heavily underappreciated because of the dismission of other body regions than the selected ones, such as the nostrils in the case of sharks. Including these often neglected organs and tissues in routine necropsies could reveal a broader range of metazoan parasites than currently recognized, highly benefiting the knowledge on general parasite biodiversity.

Among the commonly found parasite species (P > 25%) across the 3 hosts, it is noteworthy that 8 out of 9 species were not host-specific and were present in at least 2 hosts. Among these, 6 are trophically transmitted parasites, while the remaining 2 (Hexabothriidae gen. sp. and *Calicotyle* sp.) are ectoparasites found in all 3 shark species. These findings support the notion that the studied sharks are sympatric species sharing similar feeding habits, having a similar trophic position and habitat preferences (Williams et al., 1992; Klimpel et al., 2003). Parasites have also been recognized as effective indicators of hosts’ evolutionary history, with phylogenetically related host species typically sharing more parasite species (Poulin, 2010; Lima et al., 2016). Yet, the parasite community of *A. aphyodes* was more similar to that of *G. murinus* than to its congener *A. laurussonii*. This difference can be mainly attributed to the high prevalence and abundance of Cathariotrematinae gen. sp. along with the overall lower parasite burden, especially *Anisakis* Type I, observed in *A. laurussonii*. While evolutionary history is a contributing factor in shaping parasite communities, it is the ongoing ecological interactions during the species’ lifespan that most directly account for the observed patterns (Poulin, 1995).

Concerning the potential impact of parasite infections on the health condition of the studied hosts, the only significant correlation observed with the Kn was with MSR in *A. laurussonii*, suggesting that parasite burden have no major negative impact on the host’s condition. Although condition indices can fluctuate due to a variety of factors, complicating the identification of clear relationships, the long-term coevolution between parasites and sharks (Hoberg and Klassen, 2002) may have resulted in an increased host tolerance to parasitism, limiting the fitness costs of infection without necessarily preventing it (Råberg, 2014). Consistent with the present data, some studies pointed out that healthier fish often harbour more abundant and diverse parasite communities (Dallarés et al., 2014; Falkenberg et al., 2024).

The comparative data on *A. aphyodes* sampled off the west and south of Iceland revealed interesting differences in terms of parasite assemblages’ composition and structure despite the limited sample size. These differences are mainly attributed to a lower MSR and *H*, as well as higher dominance and abundance of *Anisakis* Type I in the southern sampling area. This area lies closer to the coast, with steeper topography and greater substrate heterogeneity, whereas the western sampling area is characterized by a gentler slope and more homogeneous substrate (ICES, 2022; EMODnet, 2025). The higher dominance of *Anisakis* in the southern area could potentially be linked to a preference of some cetaceans to productive coastal shelf areas (Pike et al., 2005). However, various environmental factors, along with the distribution of intermediate and definitive host species, influence small-scale spatial differences in parasite communities, making it difficult to clearly determine the causes of the observed patterns with the limited data available. Iceland is influenced by a complex system of converging oceanic water masses (Logemann et al., 2013). In this context, analysing more samples from a broader range of localities would be of great interest, as it could reveal a greater diversity of parasite species associated with these pentanchid hosts and contribute to a better understanding of the biological and ecological complexity of the region. Samples from northern Iceland would be of particular interest, since it is considered a different subarea within the Icelandic Waters ecoregion with influence of cold, low salinity Arctic waters compared to the relative warm and saline Atlantic waters influence in the southern subareas (ICES, 2022). In this sense, a preliminary study identified differences in the parasite composition of Atlantic wolffish (*Anarhichas lupus* L.) when comparing fish from the southern and northern areas (Elfarsson, 2023).

Accurately assessing parasite diversity requires consistent and thorough sampling practices. The standardized methodologies applied in this study enable a comprehensive approach and facilitate reliable comparisons. A large-scale geographic comparison is something often difficult to achieve, as many surveys either overlook specific host organs (e.g. nostrils or musculature) or concentrate solely on particular parasite groups, as explained above. Based on the joint analysis of present results with data obtained during the last years in the Mediterranean Sea by authors of the present study (Dallarés et al., 2017; Higueruelo et al., 2024), some differences in the parasite composition of catsharks with similar ecological characteristics have been detected when comparing both study areas.

The higher prevalence and abundance of nematodes in Atlantic catsharks is mainly attributed to *Anisakis* infections. In the Mediterranean Sea, *A. pegreffii* is the dominant species whereas *A. simplex*, an Arctic boreal species with a circumpolar distribution, prevails in colder waters (Mattiucci et al., 2018). Despite their different distributions, both nematode species use cetaceans as definitive hosts (Mattiucci et al., 2018). Although multiple biotic and abiotic factors influence the biogeography and infection dynamics of *Anisakis* species, the distribution and demography of their definitive hosts is a major relevant factor in explaining infection levels (Kuhn et al., 2016). The high productivity of waters around Iceland due to the confluence of warm and cold waters, among others, makes the region an important feeding ground for cetaceans (Charles et al., 2025), with 23 species recorded, of which 12 are considered regular inhabitants (Víkingsson et al., 2015). This likely contributes to the elevated *Anisakis* larval infections observed in Icelandic catsharks compared to those from the Mediterranean Sea, a pattern well documented in several teleost species (Valero et al., 2006; Levsen et al., 2018; Debenedetti et al., 2019). In contrast, the higher prevalence and abundance of crustaceans in Mediterranean catsharks is mainly attributed to the high occurrence of the copepod *Eudactylina vilelai* in *G. melastomus* (Dallarés et al., 2017), and therefore broader generalizations cannot be drawn from present results.

Regarding SACs generated in this study, the curve for *A. laurussonii*, the species with the smallest sample size, does not reach a plateau, suggesting that additional parasite species may remain undetected and that the observed diversity is likely underestimated. In addition, and consistently with previous observations on different fish species, present results indicate greater parasite species richness in Atlantic species than in their Mediterranean counterparts (Mattiucci et al., 2014; Constenla et al., 2015). Smaller fish sizes, reduced food consumptions, and lower biomass and abundance of animal communities in the Mediterranean have been proposed as potential factors contributing to this pattern (Constenla et al., 2015 and references therein). Woolley et al. (2016) found that while species richness on continental shelves and upper slopes peaks in the tropics, deep-sea species reach their highest richness at mid-to-high latitudes, particularly across the boreal Atlantic Ocean. Therefore, higher free-living species richness in these regions may lead to greater parasite richness, as more diverse host communities provide a wider range of ecological niches for parasites. This would promote host-specific adaptations and parasite speciation, resulting in more diverse parasite assemblages. Nonetheless, many biotic and abiotic factors influence the richness of parasite communities and broad generalizations must be drawn carefully.

The results presented herein highlight the potential parasite biodiversity and host–parasite relationships still to be uncovered in deepwater marine ecosystems. By shedding light on these neglected components of ecosystems, the present study contributes to the development of the growing fields of ecological and environmental parasitology. The study of parasite communities evidences the close and complex relationships occurring between parasites, their hosts and the environment, and can make a significant contribution to unravelling the intricate dynamics at play in natural systems.

## Figure 1 Long description

The map of Iceland displays sampling stations for pentanchid sharks, marked by red and blue dots. Red dots are located in the western area, while blue dots are in the southern area. The map includes depth contours labeled in meters, such as 100 m, 200 m and 500 m, indicating underwater topography. A scale bar at the bottom right shows distances up to 300 km. The map is oriented with a north arrow in the bottom left corner.

Navigate back to Figure 1.

## Figure 2 Long description

The scatter plot illustrates non-metric multidimensional scaling (nMDS) of parasite assemblages based on host species and area of capture. The horizontal axis is labeled NMDS1 and the vertical axis is labeled NMDS2. Data points are color-coded and shaped according to the host species and area: triangles for the west area and circles for the south area. The hosts are represented by different colors: A. aphyodes, A. laurussonii and G. murinus. Clusters of points indicate similarities in parasite assemblages, with notable clusters around the labels Anis, Cali, Dima, Hexa and Cath. An Euler diagram in the top left shows the distribution of parasite taxa across hosts, with overlapping areas indicating shared taxa. A Bray–Curtis similarity matrix in the top right displays mean similarities within and among hosts, with values for A. aphyodes, A. laurussonii and G. murinus. The plot reveals patterns of parasite distribution and host association, highlighting areas of dense clustering and species-specific assemblages.

Navigate back to Figure 2.

## Figure 3 Long description

Parasite species accumulation curve (P greater than 5 percent) Horizontal axis label: Individuals. Range: 0 to 40. Vertical axis label: Species richness. Range: 0 to 12. Line identification by style: Solid lines labeled: Gmur, Aaph, Alau, Scan, Gmel. Dashed lines labeled: ATL, MED. Approximate coordinate pairs read from the curves: Gmur (solid): (1, 4), (5, 8), (10, 9.5), (15, 10), (20, 10), (25, 10). ATL (dashed): (1, 3.5), (5, 7), (10, 8), (15, 8.5). Aaph (solid): (1, 3), (5, 6.5), (10, 7.5), (15, 8), (18, 8). Alau (solid): (1, 2.5), (5, 5.5), (10, 6.5), (15, 7), (18, 7). Scan (solid): (1, 2), (5, 3.5), (10, 4), (20, 4.5), (30, 5), (40, 5.2). MED (dashed): (1, 2), (5, 3.5), (10, 4), (20, 4.5), (30, 5), (40, 5.1). Gmel (solid): (1, 1.5), (5, 3.5), (10, 4), (20, 4.3), (30, 4.6), (40, 4.8). Relative levels across individuals: Gmur is highest across its plotted range, reaching about 10 species richness by about 15 to 20 individuals. ATL is below Gmur and reaches about 8.5 by about 15 individuals. Aaph is below ATL and reaches about 8 by about 18 individuals. Alau is below Aaph and reaches about 7 by about 18 individuals. Scan, MED and Gmel are lower curves that rise gradually toward about 5 to 5.2 by 40 individuals, with Gmel lowest at about 4.8 at 40 individuals.

Navigate back to Figure 3.

## Table 1 Long description

The table summarizes sample size, percent sexually mature, capture depth, total length (TL), total weight (TW), and Le Cren condition index (Kn) for three catshark species sampled in Icelandic waters, reporting means with standard deviations and ranges. Sample sizes were 17 (9 females) for A. aphyodes, 14 (2 females) for A. laurussonii, and 25 (21 females) for G. murinus. Maturity was high for A. aphyodes (94.1%) and A. laurussonii (92.9%) but lower for G. murinus (68.0%). Mean capture depth was deepest for A. laurussonii at 1112 m (range 796–1281), compared with 745 m for A. aphyodes (546–1278) and 852 m for G. murinus (466–1322). Mean TL was 67.3 cm for A. laurussonii and 60.9 cm for A. aphyodes, both longer than G. murinus at 49.5 cm; the table indicates the two Apristurus means do not differ from each other but both differ from G. murinus. Mean TW followed the same pattern (941 g A. laurussonii; 798 g A. aphyodes; 454 g G. murinus), while Kn was similar across species (about 1.01 for both Apristurus and 0.97 for G. murinus). Comparisons should be interpreted with the unequal sample sizes and sex composition across species.

Navigate back to Table 1.

## Table 2 Long description

The table reports parasite taxa found in three deepwater catsharks off Iceland, giving developmental stage, infection site, prevalence (percent infected), and mean abundance (with 95% confidence intervals when available) for each host group and capture area. Anisakis Type I (L3) occurred in multiple organs and reached 100% prevalence in all host groups; mean abundance was 28.3 in A. aphyodes (West), 68.9 in A. aphyodes (South), 4.83 in A. laurussonii (West), 71 in A. laurussonii (South), and 45.7 in G. murinus (West). Among monogeneans, Calicotyle sp. was common in A. aphyodes (West: 77.8%, MA 3.9) but low in A. laurussonii (West: 16.7%, MA 0.2) and moderate in G. murinus (West: 32.0%, MA 1.0); Hexabothriidae gen. sp. was frequent in gills, especially G. murinus (West: 96.0%, MA 5.7). Several cestodes were present mainly in A. aphyodes (West) and G. murinus (West), including Ditrachybothridium macrocephalum (66.7%, MA 3.6 in A. aphyodes West; 48.0%, MA 4.2 in G. murinus West) and Grillotia sp. (44.4%, MA 0.6 in A. aphyodes West; 36.0%, MA 0.8 in G. murinus West). Some taxa were rare and restricted to G. murinus (West), such as Anelasma squalicola (12.0%, MA 0.1) and low-prevalence copepods (each 4.0%, MA 0.04). Blank cells indicate parasites not detected or not reported for that host-area combination, and letter superscripts denote statistically significant differences among West-area host species for prevalence and/or mean abundance.

Navigate back to Table 2.

## Table 3 Long description

The table reports parasite community descriptors for several shark species, including sample size (N), total parasite richness (S), mean species richness (MSR), total mean abundance (TMA), Brillouin diversity (H), and Berger–Parker dominance (B-P dom), with 95% confidence intervals shown for most metrics when sample size exceeds two. For Iceland, A. aphyodes differs strongly by area: West has N=9 with S=8, MSR 4.3, TMA 48.0, H 0.72, and B-P dom 0.68, while South has N=8 with only one parasite species (S=1), MSR 1, H 0, and complete dominance (B-P dom 1) despite high TMA 68.9. A. laurussonii in the West (N=12) shows moderate richness and diversity (S=7; MSR 3.1; H 0.63; B-P dom 0.63), whereas the South sample is very small (N=2) with S=3, MSR 2, H 0.11, and high dominance (0.97), so comparisons there are less reliable. G. murinus in West Iceland (N=25) has the highest Icelandic richness (S=13) with MSR 4.1, TMA 59.1, H 0.59, and B-P dom 0.75. Mediterranean species have larger sample sizes but lower diversity and higher dominance overall: G. melastomus (N=120) has S=15 but low MSR 1.7 and H 0.15 with B-P dom 0.93, and S. canicula (N=61) has S=12, MSR 2.4, H 0.30, and B-P dom 0.87. Overall, Icelandic assemblages tend to have higher mean richness and diversity and lower dominance than the Mediterranean comparisons, but differences in species and sampling (including very small N for A. laurussonii South) limit direct inference.

Navigate back to Table 3.
